# A study on the mechanism affecting the Innovation and Entrepreneurship Ability of medical students based on Constructivist Theory: mediating role of Innovation and Entrepreneurship Willingness

**DOI:** 10.3389/fmed.2025.1630168

**Published:** 2025-08-29

**Authors:** Hongxu Pan, Yujia Jiang, Yu Huang, Zhihao Zhang, Junjie Du, Xuejin Song, Jianjun Zhu, Yanlin Xie, Chenchen Xu, Yong Li

**Affiliations:** ^1^School of Stomatology, Nanjing Medical University, Nanjing, Jiangsu, China; ^2^Institute of Biophysics, Chinese Academy of Sciences, Beijing, China; ^3^Department of Cardiovascular Surgery, The First Affiliated Hospital with Nanjing Medical University, Nanjing, Jiangsu, China; ^4^School of Information Science and Engineering, Southeast University, Nanjing, Jiangsu, China; ^5^Second Clinical Medical College, Nanjing Medical University, Nanjing, Jiangsu, China; ^6^First Clinical Medical College, Nanjing Medical University, Nanjing, Jiangsu, China; ^7^Fourth Clinical Medical College, Nanjing Medical University, Nanjing, Jiangsu, China; ^8^Department of Cardiology, The First Affiliated Hospital with Nanjing Medical University, Nanjing, Jiangsu, China

**Keywords:** Innovation and Entrepreneurship education, Constructivism, medical students, influence mechanism, Structural Equation Modeling

## Abstract

**Background:**

Mass Innovation and Entrepreneurship (IE) have become a global trend, yet medical education systems often fail to meet students’ innovation development needs. This study examines how universities can enhance medical students’ IE Willingness and ability.

**Methods:**

By analyzing survey data from 1,467 medical students under Constructivist Theory, we employed Amos 23.0 and SPSS 29.1 for cross-sectional path analysis. The study measured latent constructs—IE recognition, training courses, research talent models, incentive mechanisms, willingness, and ability—through 36 observed variables.

**Results:**

The analysis revealed highly significant direct effects on IE Willingness from the Research Talent Training Models (β = 0.857), IE Incentive Mechanism (β = 0.731), innovation courses (β = 0.696), and IE recognition (β = 0.521). For mediation effects on IE Ability (via willingness), only IE recognition (β = 0.122) and incentive mechanisms (β = 0.064) showed significance, while research models (β = 0.053) and innovation courses (β = 0.062) were non-significant. These findings suggest that optimizing incentive mechanisms, refining innovation courses, and strengthening research talent models—the most impactful driver—can effectively foster medical students’ innovation engagement.

## 1 Introduction

Currently, “Innovation and Entrepreneurship (IE) education” is essential for developing medical personnel adept at managing swift technology progress; yet, conventional medical education fails to meet this requirement (1). Innovation denotes the introduction of fresh ideas, methods, goods, or services aimed at substantially enhancing existing processes, technologies, or business models. Innovation encompasses not only invention but also the successful application of new ideas into practice to create value (2). IE education not only underpins the implementation of innovation-driven development strategies, but also serves as a catalyst for upgrading economic structures and enhancing the quality and efficiency of economic development (3).

However, current research exhibits significant limitations. Studies in China predominantly focus on policy and curriculum development, lacking in-depth analysis of the mechanisms influencing cognitive and motivational factors (4–6). Furthermore, weak empirical support undermines the generalizability of findings (7, 8). International research encounters significant limitations by neglecting the distinctive characteristics of the medical student demographic (9) and facing applicability challenges of models from developed nations (10–12).

Specifically, China employs a “policy-hospital-university” cooperation framework; medical innovation must conform to the “Healthy China 2030” Plan Outline (13), prioritizing pilot promotion within medical consortia and coordinated approval for medical insurance coverage (14). In North America and Europe, as illustrated by Stanford’s Biodesign, prioritizes the “clinical need identification-market translation” pathway (15, 16), fundamentally relying on collaboration between physicians and engineers, alongside venture capital-driven methodologies (16–18). The Mayo Clinic’s SPARC framework employs a sophisticated interdisciplinary collaboration system, incorporating physicians, engineers, and designers, to establish an agile “problem-solution-validation” loop. It also utilizes advanced technology transfer mechanisms and venture capital networks to effectively link innovation outcomes to commercialization pathways (19, 20).

The differing perspectives on IE education between China and the West have prompted us to reflect on how to enhance higher education, particularly the IE competencies of medical students. Empirical studies demonstrate that Constructivism has exerted guiding influence across diverse educational domains including clinical medicine, teacher training, nursing, music pedagogy, and dancing (21–25). It provides a theoretical framework for creating multidimensional, open learning environments, enhances educational experiences through team-based collaborative learning, and promotes interdisciplinary shifts in teaching paradigms. However, few studies have applied Constructivism to IE education, limiting its conceptual depth and expansibility.

Overall, there exists two significant research gap in current medical education: (1) existing studies demonstrate a notable deficiency in exploring medical students’ Innovation and Entrepreneurship capabilities, as traditional medical education overemphasizes clinical skills training while neglecting the cultivation of innovative thinking and entrepreneurial literacy (26) - a situation that has become increasingly inadequate to meet the demands for interdisciplinary medical talents in the context of rapid medical technology advancement and healthcare industry transformation; (2) few studies have treated entrepreneurial willingness, which reflects students’ psychological preparedness for innovation activities, as a key predictive variable for medical students’ Innovation and Entrepreneurship capabilities, and overlooking this subjective initiative’s driving role in competency development would hinder our comprehensive understanding of how Innovation and Entrepreneurship education influences medical students’ ability development.

Therefore, by investigating the impact mechanism of Innovation and Entrepreneurship education on medical students’ IE Ability, particularly revealing the mediating role of IE Willingness, this study will not only enrich the theoretical framework of medical education but also provide scientific evidence for developing targeted Innovation and Entrepreneurship training programs, making significant contributions to advancing the reform of medical talent cultivation models under the New Medical Education initiative.

## 2 Literature review and research hypotheses

### 2.1 IE education from a Constructivist perspective

In contrast to Social Cognitive Theory (which emphasizes skill acquisition through observation and imitation) (27), Self-Determination Theory (which highlights motivation driven by psychological needs) (28, 29), or Experiential Learning (which focuses on the experience cycle) (30–33), the distinctive merit of Constructivist Learning Theory resides in its significant elucidation of how “active knowledge construction” concurrently enhances higher-order cognitive abilities and fosters innovation motivation: In IE education, students utilize prior experiences to analyze, evaluate, and reconstruct new knowledge (e.g., principles of innovation), thereby cultivating essential skills such as problem identification, solution generation, and critical decision-making; this enhancement of capabilities, in turn, intrinsically bolsters their motivation to address complex real-world challenges. Constructivism concurrently underscores the development of self-regulatory skills, empowering students to proactively adjust to the evolving nature of the innovation process and maintain motivation—illuminating the inherent mechanisms of “ability-motivation mutual reinforcement” and “dynamic environment adaptation” inadequately addressed by alternative theories.

Constructivism asserts that learning is an active process in which pupils build knowledge using prior experiences and cognitive frameworks, rather than passively absorbing information (34, 35). In IE education, active learning facilitates students’ understanding of innovation principles and their application to actual problem-solving, hence improving both skills and motivations. This concept aligns with the fundamental tenets of Constructivism, which emphasizes cognitive competencies—including analytical, evaluative, and critical thinking skills—that empower students to identify problems, devise innovative solutions, and make informed decisions in complex environments.

### 2.2 IE education and IE Willingness

The complex process of developing medical expertise is always faced with new demands due to the growing issues of global health (36). According to Piaget, Constructivism places a strong emphasis on active learning, where students build and deepen their comprehension by drawing on their unique perspectives and experiences (37). The Chinese government has continuously placed a high priority on entrepreneurial education since 1996, with an emphasis on encouraging it from a variety of angles within academic institutions (38). As stated in the 2010 Ministry of Education, The Ministry of Science and Technology’s Measures Identified about Practice Base for Science and Technology Enterprise for College Students, the goal of improving innovative entrepreneurship education is to increase students’ awareness of entrepreneurship, encourage entrepreneurial activities, and cultivate an innovative spirit in them (39). Graduate students’ innovative and practical skills can be significantly improved by reforms and innovations made to the tutor team’s development, practice bases, and talent training models (40). Based on Constructivism, we put forth hypothesis

IE education has a positive influence on IE Willingness.

### 2.3 IE education and IE Ability

Students’ attention and involvement are the cornerstones of constructive education, and they can only be assisted in completing the active construction of knowledge by encouraging their active engagement, which in turn enhances their capacity for innovation (41). In addition to methodically teaching students the knowledge and abilities required for IE, the scientific Research Talent Training Models and customized Innovation Talent Training Courses, also integrate theoretical knowledge with real-world scenarios. By strengthening their creative thinking and problem-solving skills, this method helps students better comprehend and deal with the difficulties they face during the IE process, thereby increasing their total IE Ability (42). Good reward programs can also inspire students’ inventiveness and enthusiasm for learning while promoting their active participation in practice. Thus, the study proposes the hypothesis

IE education has a positive influence on IE Ability.

### 2.4 The mediating role of IE Willingness

After decades of constant development, IE education in Chinese colleges and universities has advanced to an intermediate stage, depending on student participation as well as the school’s knowledge and resources as a guide for the main subject. Constructivist Theory views education as a dynamic process. It is not possible to inject or absorb information into students since they are not empty vessels. Students must engage with real-world examples and experiences in order to develop the meaning of the course material (43). Enhancing students’ proactive involvement is therefore particularly important since it marks a big shift from students’ passive response to active production. Thus, we put forth the following hypothesis,

IE Willingness mediates between IE education and IE Ability.

### 2.5 Model construction

Based on the above analysis and research hypotheses, we have developed a research model with Innovation and Entrepreneurship education as the antecedent variable, students’ IE Ability as the outcome variable, and IE Willingness as the mediating variable. Appendix 1 lists the exact components of the 31 measurement items that constitute the model.

## 3 Research methods

### 3.1 Study design

**FIGURE 1:**
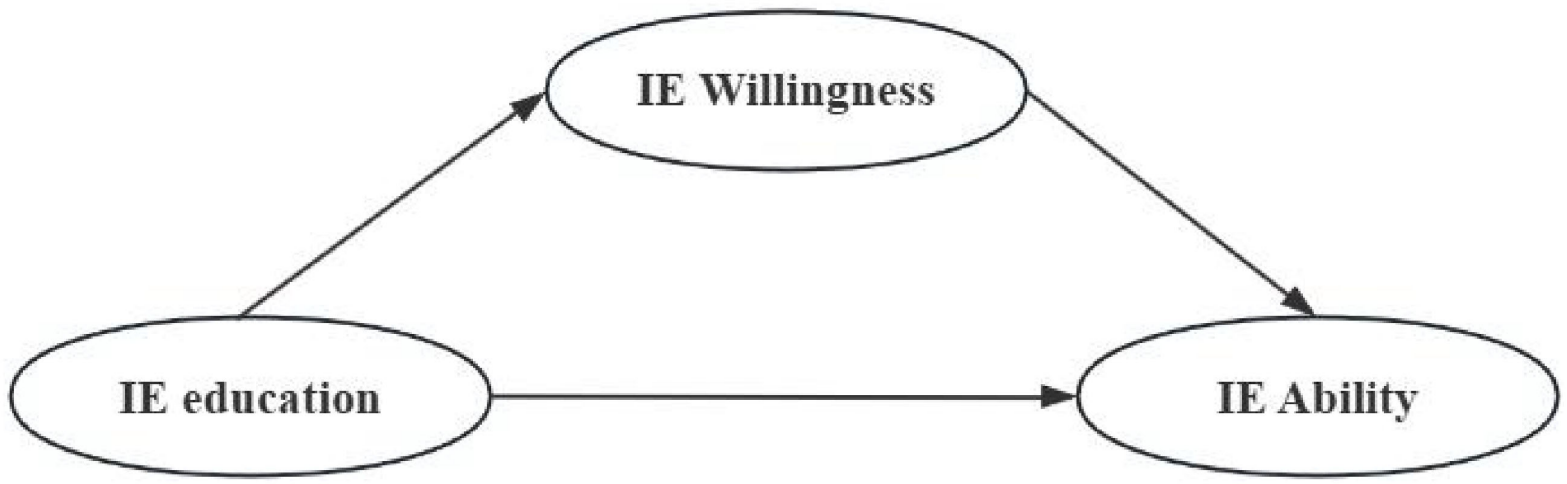
The initial hypothetical model.

The present study utilized Structural Equation Modeling (SEM) to analyze the data, chosen for its ability to concurrently assess direct and indirect effects, accommodate latent variables with measurement error, and provide superior performance compared to traditional regression techniques in complex path analyses. Specifically, this study first employed SPSS 22 software to conduct exploratory factor analysis (EFA) and confirmatory factor analysis (CFA) to identify the specific items corresponding to each dimension. Subsequently, composite reliability (CR), average variance extracted (AVE), and Pearson correlation coefficients were calculated to examine the scale’s reliability and validity. After confirming that the model construction requirements were met, Amos 23.0 software was used to establish the structural equation model illustrated in Figure 1, followed by a comprehensive evaluation of the model’s goodness-of-fit, with *p* < 0.05 (44).

### 3.2 Data collection

Data for this study was gathered using a questionnaire survey. The details of the specific questionnaire can be found in the Supplementary materials. An online survey was disseminated to 35 medical schools in Eastern China, including “Double First Class” medical universities, ordinary undergraduate medical schools, and specialized vocational medical colleges, as part of a cross-sectional study design. The exclusion criteria for invalid questionnaires in our study included: (1) self-contradictory answers with obvious logical conflicts, (2) unusual response patterns such as selecting the same answer for all questions, (3) missing mandatory items (e.g., institution of study), and (4) response times exceeding 5 min (indicating excessive deliberation) or falling below 60 s (suggesting inattentive responses). Out of the 1,879 questionnaires collected, 1,467 were deemed valid, yielding a response rate of 78.07%.

### 3.3 Measurements

#### 3.3.1 IE education

Drawing on A’s research, this study constructs an Innovation and Entrepreneurship education system comprising four interconnected and distinctive sub-dimensions: (1) IE Recognition Dimension, which examines the awareness level of university faculty, students, and administrators regarding the value of Innovation and Entrepreneurship education, aiming to enhance institutional participation through campus culture development and policy advocacy; (2) Innovative Talent Cultivation Curriculum Dimension, evaluating the impact of specialized courses such as interdisciplinary workshops and business model design on students; (3) Research-Oriented Talent Development Model Dimension, assessing programs like undergraduate research training initiatives and patent commercialization projects in medical universities to facilitate the transformation of academic achievements into practical applications; and (4) IE Incentive Mechanism Dimension, measuring the effectiveness of diversified incentive systems including startup funding, credit substitution policies, and competition rewards.

#### 3.3.2 IE Willingness

Innovation and Entrepreneurship Willingness consists of several key dimensions, reflecting learners’ recognition of the multidimensional value of IE education (45). These characteristics include: the need for academic ability enhancement (e.g., clinical research skills, academic performance), the expectation of further education support (e.g., postgraduate entrance exam preparation), the pursuit of improved employability, and the emphasis on expanding global perspectives. The intensity of this willingness is shaped by individual goals and needs in academic development, career planning, and global competency.

#### 3.3.3 IE Ability

Innovation and Entrepreneurship Ability is reflected in the degree of knowledge transformation (46) and consists of seven key components. These components are primarily manifested through students’ acquisition of innovation methodologies and entrepreneurial process knowledge via systematic training programs (such as IE-themed camps), the development of research capabilities and business thinking through practical platforms, as well as the enhancement of project design and presentation skills by participating in various competitions, including the “Challenge Cup” and “Internet Plus” contests.

### 3.4 Ethical approval

The Ethics Committee of Nanjing Medical University granted approval for this study under approval number “Nanjing Medical University Ethics Review (2025) No.4.” Because the survey was anonymous, all participants were guaranteed to be aware of the goals and methods of the study before taking part. The information was kept private and exported in Microsoft Office Excel’s.xlsx format.

### 3.5 Participants

The study sample consisted of 1,467 medical students with distinct demographic characteristics. Gender distribution showed a slightly higher proportion of female students (819, 55.8%) compared to male students (648, 44.2%). In terms of academic standing, the sample was predominantly composed of early-stage medical students, with first- and second-year undergraduates accounting for the vast majority (1,382, 94.2%). Advanced undergraduates (Years 3–5) and graduate students were minimally represented, comprising less than 2% each (Year 3–4: 28, 1.9%; Year 5: 23, 1.6%; Master’s: 18, 1.2%; Doctorate: 16, 1.1%) (Table 1). This distribution suggests that the findings may be particularly reflective of early medical education experiences, while the perspectives of more advanced and research-focused students may require further investigation. The relatively balanced gender distribution and pronounced concentration of early-stage students should be considered when interpreting and generalizing the study results.

**TABLE 1:** Demographic characteristics of participants.

Variables	Classification	Number	%
Gender	Male	648	44.2
	Female	819	55.8
Academic year	Year 1–2	1,382	94.2
	Year 3–4	28	1.9
	Year 5	23	1.6
	Master’s	18	1.2
	Doctorate	16	1.1

## 4 Results

### 4.1 Common method biases (CMB)

To examine potential common method biases (CMB) in respondents’ answers, this study conducted Harman’s single-factor test using SPSS 29.0. The results showed that a single factor accounted for 33.418% of the variance, which is below the critical threshold of 40%, indicating no significant common method bias in this study.

### 4.2 Reliability and validity test

In psychological research, Cronbach’s alpha coefficient and factor analysis are commonly employed to assess questionnaire reliability and the replicability of experimental results. Specifically, Cronbach’s alpha is used to measure questionnaire reliability, while exploratory factor analysis examines the underlying relationships among variables. As shown in Table 2, all items in this study demonstrated Cronbach’s alpha values exceeding 0.75, with factor loadings for individual items and KMO values for all six factors surpassing 0.65. Comprehensive analysis indicates that the six-factor model demonstrates good fit, meeting the construct validity criteria proposed by Fornell and Larcker (47).

**TABLE 2:** Construct validity.

Variable	Cronbach’s alpha	Factor loading	KMO
Recognition of IE	0.710	0.754–0.786	0.726
IE Incentive Mechanism	0.822	0.628–0.803	0.893
Innovation Talent Training Courses	0.874	0.712–0.820	0.728
Research Talent Training Models	0.939	0.722–0.829	0.892
IE Willingness	0.975	0.714–0.881	0.911
IE Ability	0.627	0.707–0.815	0.831

### 4.3 Validity and reliability analysis

This study conducted validation analyses using Composite Reliability (CR), Average Variance Extracted (AVE), and the square roots of AVE for each questionnaire dimension. Higher CR and AVE values indicate better internal consistency, reliability, and explanatory power of the measurement model. As shown in Table 3, all dimensions demonstrated CR values exceeding 0.7, meeting acceptable standards. The diagonal values in Table 3 represent the square roots of AVE for each factor, reflecting the strength of within-factor correlations, while off-diagonal values indicate between-factor correlations. According to Fornell and Larcker’s (47) discriminant validity criterion, when the square root of AVE exceeds the absolute value of correlations between latent variables, it demonstrates stronger within-factor than between-factor correlations, indicating good discriminant validity (47). Table 3 confirms that the questionnaire in this study meets all requirements for validity and reliability analysis.

**TABLE 3:** Validity and reliability analysis.

Variable	CR	AVE	1	2	3	4	5	6
1. Recognition of IE	0.718	0.501	**0.862**					
2. IE Incentive Mechanism	0.721	0.558	0.709	**0.781**				
3. Innovation Talent Training Courses	0.829	0.553	0.083	0.250	**0.776**			
4. Research Talent Training Models	0.809	0.540	0.178	0.195	0.054	**0.823**		
5. IE Willingness	0.831	0.492	0.218	0.447	0.347	0.419	**0.680**	
6. IE Ability	0.806	0.518	0.317	0.271	0.288	0.248	0.180	**0.701**

The numbers on the diagonal of Table 3 (in bold) are the square roots of the AVE values for each factor, used to indicate the strength of the internal correlations within the factors, while the remaining numbers represent the correlations between factors.

### 4.4 Model fit analysis

The analysis of model fit was conducted using Amos 23.0. According to the investigation’s findings, the model fits well and is appropriate for regression analysis. Table 4 presents the specifics.

**TABLE 4:** Model fit parameters.

Fit index	χ^2^/df	SRMR	RMSEA	GFI	AGFI	NFI	TLI	CFI
Fit criteria	< 3	< 0.08	< 0.08	> 0.90	> 0.90	> 0.90	> 0.90	> 0.90
Calculated results	2.656	0.034	0.044	0.929	0.911	0.953	0.959	0.966

SRMR, Standardized Root Mean Square Residual; RMSEA, Root Mean Square Error of Approximation; GFI, Goodness-of-Fit Index; AGFI, Adjusted Goodness-of-Fit Index; NFI, Normed Fit Index; TLI, Tucker-Lewis Index; CFI, Comparative Fit Index.

### 4.5 Effect value analysis

Amos 23.0 determined the causal relationship between variables. The results demonstrate that the coefficients for students’ IE Willingness in the direct path impact test were all positive and statistically significant at the 0.001 level for the Recognition of IE, Innovation Talent Training Courses, Research Talent Training Models, and IE Incentive Mechanism. This supports Hypothesis H1 by showing that these elements significantly improve the quality of IE instruction for clinical medical students. At the 0.05 significance level, all four characteristics demonstrated positive and significant regression coefficients for students’ IE Ability, indicating a beneficial impact on clinical medical students’ IE Ability and supporting Hypothesis H2.

The indirect route effect test, significant at the 0.05 level, demonstrated that students’ Recognition of IE and the IE Incentive Mechanism affected their IE ability, with this relationship mediated by their propensity to innovate and engage in entrepreneurship. The mediation role of IE Willingness in the relationships between students’ IE Ability and the Innovation Talent Training Courses, as well as the Research Talent Training Models, was relatively minor, with significant levels of 0.289 and 0.119, respectively. Consequently, only a portion of Hypothesis H3 was validated. Table 5 presents the findings.

**TABLE 5:** Regression analysis results.

Path	β	*P*-value	Decision
IE Incentive Mechanism→IE Willingness	0.731	< 0.001	Supported
Recognition of IE→IE Willingness	0.521	< 0.001	Supported
Research Talent Training Models→IE Willingness	0.857	< 0.001	Supported
Innovation Talent Training Courses→IE Willingness	0.696	< 0.001	Supported
IE Incentive Mechanism→IE Ability	0.064	0.017	Supported
Recognition of IE→IE Ability	0.122	< 0.001	Supported
Research Talent Training Models→IE Ability	0.053	0.048	Supported
Innovation Talent Training Courses→IE Ability	0.062	0.023	Supported
IE Incentive Mechanism→IE Willingness→IE Ability	0.068	0.011	Supported
Recognition of IE→IE Willingness→IE Ability	0.136	< 0.001	Supported
Research Talent Training Models→IE Willingness→IE Ability	0.056	0.289	–
Innovation Talent Training Courses→IE Willingness →IE Ability	0.059	0.119	–

## 5 Discussion

This study, grounded in Constructivist Theory, demonstrates significant advantages in fostering students’ IE intention and competencies through mechanisms such as IE incentive systems, IE cognition, research-oriented talent cultivation models, and IE talent development curricula. These factors provide students with fundamental motivation and support to participate in IE activities and enhance their skills.

The mediating role of IE Willingness appears to be minimal in the influence pathways between Research Talent Training Models and Innovation Talent Training Courses regarding the IE Ability of clinical medical students. This outcome may be attributed to several factors. A comprehensive analysis can identify the following causes. The main limitation of the Research Talent Training Models is the insufficient integration of clinical practice with research (48). The activation of IE Willingness is hindered by students’ difficulty in applying research findings to clinical practice. Despite recent increases in funding and improvements in instruction for IE education, the growing student population indicates that the available resources for high-quality clinical medical training and research are insufficient to meet all student needs. The development of students’ IE willingness is significantly influenced by the lack of sufficient practical opportunities.

While some courses related to Innovation and Entrepreneurship have been introduced, the majority of Innovation Talent Training Courses continue to focus on traditional medical theory and clinical practice (49). The development of IE theory and practical skills remains relatively limited. Encouraging student enthusiasm for entrepreneurship is difficult due to the course material’s deficiencies in creativity, practicality, interaction, and discussion. Furthermore, each student possesses distinct skills, and their engagement with and receptiveness to IE varies. Some students who are more traditional or insecure may find it more challenging to develop a strong enthusiasm for using IE.

The research findings reveal significant structural differences in comparison to both global and Chinese studies. Internationally, Western models, such as U.S. biodesign programs, hold the view that the high level of interest and participation in the program highlight the need for incorporating innovative training in UME to foster creativity and prepare future physicians to contribute to the advancements in healthcare (50). In contrast, this study reveals that within China’s resource-constrained medical education context, direct structural interventions, especially research talent cultivation models (β = 0.857), bypass intention mediation and have a more pronounced impact on ability development.

In China, previous studies emphasize policy-level drivers (51) or theoretical curriculum reforms. This study empirically deconstructs how specific pedagogical antecedents, such as the “IE Incentive Mechanism” and “Recognition of IE,” differentially activate medical students’ IE pathways. We identify a significant misalignment: “Top Medical Talent” programs (β = 0.250) surpass other domestic training initiatives, such as Anhui Medical University’s passive career-alternative models. However, their limited mediation through IE willingness (β = 0.056, *p* = 0.289) indicates systemic deficiencies in translating intention into ability, a challenge not previously addressed in the literature.

Our analysis revealed that the mediating effect in the two pathways, “Research Talent Training Models → IE Willingness → IE Ability” (β = 0.056, *P* = 0.289) and “Innovation Talent Training Courses → IE Willingness → IE Ability” (β = 0.059, *P* = 0.119), was comparatively weak. Despite over 90% approval for various “Research Talent Training Models,” the availability of high-quality clinical resources and research platforms is limited, failing to meet student needs and hindering the activation of their Innovation and Entrepreneurship intentions. These training models are challenging to access for most students who intend to innovate and start businesses in the current context. Moreover, “Direct Admission to Graduate Programs” and “Additional Credits for Compulsory Courses” exerted significantly greater influence on students’ intentions regarding IE than on their capabilities in IE. This discrepancy may arise from measurement bias, temporal lag effects, and hindered transformation pathways from intention to ability.

In the latter path, only 49.87% of students strongly supported the integration of clinical scenarios in innovation courses, contrasting sharply with the greater practical alignment noted in research-oriented models. This indicates that students exhibit low acceptance of IE, subsequently impacting the efficiency of their willingness to convert. Additionally, around 10% of students remain opposed to different types of “Innovation Talent Training Courses.” The course content has historically favored traditional medical theories, resulting in a deficiency of innovative practices and interactive discussions. This has resulted in a student mindset that is hesitant to adopt new course models. Moreover, igniting students’ creativity and entrepreneurial skills is challenging due to the course material’s lack of originality and practical relevance, coupled with insufficient interaction and discussion. The existing curricula place excessive focus on conventional medical theories, neglecting IE theoretical frameworks and practical competencies, which results in insufficient student engagement and participation.

## 6 Conclusion

This study, rooted in Constructivist Theory—which asserts that contextualized practice is fundamental to knowledge construction, social interaction fosters competency development, and learner initiative is crucial for transforming intention into capability—systematically explores the mechanisms involved in the formation of intention and capability within medical Innovation and Entrepreneurship education.

Diverse measures within the four components of the IE Incentive Mechanism, Recognition of IE, Research Talent Training Models, and Innovation Talent Training Courses have varying degrees of beneficial impact on the enhancement of students’ Innovation and Entrepreneurship abilities. The Research Talent Training Models shown markedly greater direct effects on IE Ability (β = 0.857, *p* < 0.001) compared to other variables, such as IE Incentive Mechanism (β = 0.731, *p* < 0.001) and Innovation Talent Training Courses (β = 0.696, *p* < 0.001). The “direct enrolment of graduate students” and “additional credits for required courses” significantly enhance students’ interest and passion in IE, hence fostering their willingness to participate and their developmental abilities. The comprehension of policy in IE cognition had the highest efficacy. Innovative talent programs, such as PBL, can proficiently enhance students’ research and innovation skills. Within the variables related to Research Talent Training Models, the “Top Medical Talent” Education Plan placed top in its category, providing promising avenues for the optimization of future talent development systems. The aforementioned measures can indirectly enhance the levels of Innovation and Entrepreneurship by augmenting students’ propensity to develop and establish enterprises. From a constructivist viewpoint, enhancing student acceptance of IE should be prioritized. Students are urged to proactively pursue knowledge, question assumptions, and engage in critical thinking; in other words, they are active makers of knowledge.

## 7 Limitations and future research

This study has limitations even if it provides insightful information about the factors influencing medical students’ IE skills. The study sample primarily consisted of undergraduate students from 35 medical colleges in Eastern China, with low participation rates among graduate students, which may limit the generalization of conclusions to higher-degree populations. Moreover, The study’s relevance to other disciplines and systems is limited by its concentration on Chinese medical schools during a certain period, and since IE education is dynamic, changes may take place.

Therefore, in future research, it is recommended to expand the sample range and diversity, optimize data collection and measurement tools, increase the multi-dimensional measurement of the questionnaire on curriculum design, teaching methods and student background, and adopt experimental design or quasi-experimental design to evaluate the effectiveness of IE education measures. In addition, it is suggested to conduct longitudinal studies to track the changes in students’ abilities at different educational stages and reveal the long-term impact of educational intervention.

## Data Availability

The raw data supporting the conclusions of this article will be made available by the authors, without undue reservation.

## Appendix

**APPENDIX 1:** Variables and measurement items.

Variables	Measurement items
IE Incentive Mechanism	Award of “Personal Honor”
	Priority consideration for “Recommended Excellence”
	“Direct Admission to Graduate Programs”
	“Additional Credits for Compulsory Courses”
	“Financial Rewards”
	“Support from Mentors”
Recognition of IE	Understanding of support policies
	Understanding of specific details of IE education
	Support for IE education for medical students
	Support for IE courses
	Recognition and balance of IE courses with medical professional studies
Research Talent Training Models	“Integrated Undergraduate and Graduate” training model
	“Industry-Academia-Research Integration” training model
	“Institute-to-Institute” integrated training model
	Clinical phase training model for general practitioners
	“Top Medical Talent” education plan
Innovation Talent Training Courses	Offering of “IE” elective courses (e.g., basic medical entrepreneurship)
	“Medical PBL Case Studies” and other required IE courses
	“Second Classroom” and other medical innovation practice activities
IE Willingness	IE education helps improve comprehensive clinical research ability
	IE education enhances academic performance
	IE education contributes to further studies for postgraduate entrance examinations
	IE education improves employment quality
	IE education enhances global perspective
IE Ability	Outstanding participant in college ie training camps
	“Seminar on IE”
	College IE training program
	“Undergraduate Mentor Program”
	Awards in “Challenge Cup” series competitions
	Awards in “Internet+” series competitions
	Other IE competition awards
