# Supplementary material for: A study on the mechanism affecting the Innovation and Entrepreneurship Ability of medical students based on Constructivist Theory: mediating role of Innovation and Entrepreneurship Willingness
Source: Front Med (Lausanne). 2025 Aug 29;12:1630168. doi: 10.3389/fmed.2025.1630168 (PMC12426197; doi:10.3389/fmed.2025.1630168)
Supplement: Supplementary file 1 [file Data_Sheet_1.pdf]

**Dear Respondents,**

**Hello! Thank you very much for taking the time to participate in this survey. Your answers will be of great help to our research. All information is for statistical analysis only, please feel free to answer truthfully.**

Part I: IE Incentive Mechanism

a1.Does the incentive mechanism for awarding "personal honor" in the school's innovation and entrepreneurship education help improve your enthusiasm for participating in innovation and entrepreneurship courses?

A.Strongly Disagree B.Disagree C.Neutral D.Agree E.Strongly Agree

a2. Does the incentive mechanism for priority consideration for "Recommended Excellence" in the school's innovation and entrepreneurship education help improve your enthusiasm for participating in innovation and entrepreneurship courses?

A.Strongly Disagree B.Disagree C.Neutral D.Agree E.Strongly Agree

a3. Does the mechanism of direct admission to postgraduate studies in the

school's innovation and entrepreneurship education help improve your enthusiasm for participating in innovation and entrepreneurship?

A.Strongly Disagree B.Disagree C.Neutral D.Agree E.Strongly Agree

a4.Does the mechanism of "Additional Credits for Compulsory Courses" in the school's innovation and entrepreneurship education help improve your enthusiasm for participating in innovation and entrepreneurship?

A.Strongly Disagree B.Disagree C.Neutral D.Agree E.Strongly Agree

a5. Does the mechanism of "Financial Rewards" in the school's innovation and entrepreneurship education help improve your enthusiasm for participating in innovation and entrepreneurship?

A.Strongly Disagree B.Disagree C.Neutral D.Agree E.Strongly Agree

a6. Does the mechanism of "Support from Mentors" in the school's innovation and entrepreneurship education help improve your enthusiasm for participating in innovation and entrepreneurship?

A.Strongly Disagree B.Disagree C.Neutral D.Agree E.Strongly Agree

Part II: Recognition of IE

b1. Are you familiar with the national support policy documents for "organizing and carrying out innovation and entrepreneurship education" in our country?

A.Strongly Disagree B.Disagree C.Neutral D.Agree E.Strongly Agree

b2. Are you familiar with specific details of IE Education in our country?

A.Strongly Disagree B.Disagree C.Neutral D.Agree E.Strongly Agree

b3. Are you familiar with support for IE Education for medical students?

A.Strongly Disagree B.Disagree C.Neutral D.Agree E.Strongly Agree

b4. Are you familiar with support for IE Courses?

A.Strongly Disagree B.Disagree C.Neutral D.Agree E.Strongly Agree

b5. Do you know how to balance between IE courses and medical

professional studies?

A.

Part III: Research Talent Training Models

c1. Do you think the "Integrated Undergraduate and Graduate" training model is conducive to IE education?

A.Strongly Disagree B.Disagree C.Neutral D.Agree E.Strongly Agree

c2. Do you think the "Industry-Academia-Research Integration" training model is conducive to IE education?

A.Strongly Disagree B.Disagree C.Neutral D.Agree E.Strongly Agree

c3. Do you think the "Institute-to-Institute" training model is conducive to IE education?

A.Strongly Disagree B.Disagree C.Neutral D.Agree E.Strongly Agree

c4. Do you think the clinical phase training model for general practitioners is conducive to IE education?

A.Strongly Disagree B.Disagree C.Neutral D.Agree E.Strongly Agree

c5. Do you think the "Top Medical Talent" education plan is conducive to IE education?

A.Strongly Disagree B.Disagree C.Neutral D.Agree E.Strongly Agree

Part IV: Innovation Talent Training Courses

d1. Do you think offering of "IE" elective courses (e.g., Basic Medical Entrepreneurship) is suitable as a content form for IE education?

A.Strongly Disagree B.Disagree C.Neutral D.Agree E.Strongly Agree

d2. Do you think "Medical PBL Case Studies" and other required IE courses is suitable as a content form for IE education?

A.Strongly Disagree B.Disagree C.Neutral D.Agree E.Strongly Agree

d3. Do you think "Second Classroom" and other medical innovation practice activities is suitable as a content form for IE education?

A.Strongly Disagree B.Disagree C.Neutral D.Agree E.Strongly Agree

Part V: IE Willingness

e1. Do you agree that IE education helps improve comprehensive clinical research ability?

A.Strongly Disagree B.Disagree C.Neutral D.Agree E.Strongly Agree

e2. Do you agree that IE Education enhances academic performance?

A.Strongly Disagree B.Disagree C.Neutral D.Agree E.Strongly Agree

e3. Do you agree that IE Education contributes to further studies for postgraduate entrance examinations?

A.Strongly Disagree B.Disagree C.Neutral D.Agree E.Strongly Agree

e4. Do you agree that IE Education improves employment quality?

A.

e5. Do you agree that IE Education enhances global perspective?

A.Strongly Disagree B.Disagree C.Neutral D.Agree E.Strongly Agree

Part VI: IE Ability

f1. Have you been awarded with “Outstanding Participant” in College IE Training Camps?

A.Strongly Disagree B.Disagree C.Neutral D.Agree E.Strongly Agree

f2. Have you participated in "Seminar on IE"?

A.Strongly Disagree B.Disagree C.Neutral D.Agree E.Strongly Agree

f3. Have you participated in College IE Training Program?

A.Strongly Disagree B.Disagree C.Neutral D.Agree E.Strongly Agree

f4. Have you participated in "Undergraduate Mentor Program"?

A.Strongly Disagree B.Disagree C.Neutral D.Agree E.Strongly Agree

f5. Have you won awards in "Challenge Cup" series competitions?

A.Strongly Disagree B.Disagree C.Neutral D.Agree E.Strongly Agree

f6. Have you won awards in "Internet+" series competitions?

A.Strongly Disagree B.Disagree C.Neutral D.Agree E.Strongly Agree

f7. Have you won awards in other IE competitions?

A.Strongly Disagree B.Disagree C.Neutral D.Agree E.Strongly Agree
